# Studying Fungal-Bacterial Relationships in the Human Gut Using an In Vitro Model (TIM-2)

**DOI:** 10.3390/jof9020174

**Published:** 2023-01-28

**Authors:** Evy Maas, John Penders, Koen Venema

**Affiliations:** 1 Centre for Healthy Eating and Food Innovation, Maastricht University–Campus Venlo, Villafloraweg 1, 5928 SZ Venlo, The Netherlands; 2 Euregional Microbiome Center, P. Debyelaan 25, 6229 HX Maastricht, The Netherlands; 3 Department of Medical Microbiology, School of Nutrition and Translational Research in Metabolism (NUTRIM) and Care and Public Health Research Institute (Caphri), Maastricht University, P. Debyelaan 25, 6229 HX Maastricht, The Netherlands

**Keywords:** gut microbiota, fungi, bacteria, in vitro model, cross-kingdom relations

## Abstract

The complex microbial community found in the human gut consist of members of multiple kingdoms, among which are bacteria and fungi. Microbiome research mainly focuses on the bacterial part of the microbiota, thereby neglecting interactions that can take place between bacteria and fungi. With the rise of sequencing techniques, the possibilities to study cross-kingdom relationships has expanded. In this study, fungal-bacterial relationships were investigated using the complex, dynamic computer-controlled in vitro model of the colon (TIM-2). Interactions were investigated by disruption of either the bacterial or fungal community by the addition of antibiotics or antifungals to TIM-2, respectively, compared to a control without antimicrobials. The microbial community was analyzed with the use of next generation sequencing of the ITS2 region and the 16S rRNA. Moreover, the production of SCFAs was followed during the interventions. Correlations between fungi and bacteria were calculated to investigate possible cross-kingdom interactions. The experiments showed that no significant differences in alpha-diversity were observed between the treatments with antibiotics and fungicide. For beta-diversity, it could be observed that samples treated with antibiotics clustered together, whereas the samples from the other treatments were more different. Taxonomic classification was done for both bacteria and fungi, but no big shifts were observed after treatments. At the level of individual genera, bacterial genus *Akkermansia* was shown to be increased after fungicide treatment. SCFAs levels were lowered in samples treated with antifungals. Spearman correlations suggested that cross-kingdom interactions are present in the human gut, and that fungi and bacteria can influence each other. Further research is required to gain more insights in these interactions and their molecular nature and to determine the clinical relevance.

## 1. Introduction

In the past decades, the development of techniques for the analysis of the microbial community in the human gut has led to new insights in this ecosystem. The use of next-generation sequencing allowed the study of microorganisms that are culture-independent and showed the complexity of the gut microbiota [1]. The majority of research on the human gut microbiota is focused on the bacterial component, but the use of sequencing techniques has also shown that fungi are an important component of the microbiota [2]. Several studies have described the gut fungal community, also named mycobiome, and how these can be linked to several diseases, not only gastrointestinal (GI), but also metabolic diseases [3,4,5,6]. Both bacteria and fungi can be influenced by external factors, such as diet or antibiotic use [7,8,9]. Since these two communities share the same environment, the influence on one kingdom is hypothesized to also lead to a change in the other. These fungal-bacterial cross-kingdom interactions are largely unknown, and with that, also the influence of these interactions on host health.

An example of a disturbance of these fungal-bacterial interactions is the disruption of the bacterial community by antibiotic use that can lead to dysbiosis, which can subsequently lead to the overgrowth of opportunistic pathogenic fungi, such as *Candida* [10]. The development of antibiotic-induced dysbiosis has been described before [11]. The removal of not only pathogenic microbes but also commensals by antibiotic use has led to a disruption of the complex microbial community, which can lead to risks for the human health, such as *Clostridioides difficile* infection after antibiotic use [12]. The widespread use of antibiotics has also led to a rise in fungal infections that occur after this treatment. These fungal infections are often difficult to treat and have a high mortality rate [13]. Similarly, treatment with anti-fungal treatment leads to disruptions in the bacterial community [14]. More information on how these communities interact and how this is influenced when one or the other community is disrupted can possibly aid in the prevention of some fungal infections that are a problem, particularly in e.g., hospitalized or immunocompromised patients.

The use of sequencing techniques has helped in the study of the microbial community in the gut, but there are still challenges. The diet is an important source of fungi, and it is not always clear if the fungi found through sequencing of fecal samples are fungal species that can colonize the gut or are transient species from the diet that only pass through the GI tract [15]. This makes it difficult to interpret results and to show interactions between bacteria and fungi. A tool that can give additional information on these two kingdoms within the gut microbiota and how these are modulated is an in vitro model [16]. There are several examples of in vitro models of the GI tract (stomach, small intestine, and large intestine). The majority of the microbial community in the gut is found in the large intestine, and therefore the complex computer-controlled in vitro model of the colon (TIM-2) was used in this study to investigate the bacterial and fungal communities and how these interact [17]. The TIM-2 system has been used previously for the study of the bacterial microbiota in the human colon [18,19]. The model allows for the control of environmental factors such as temperature, pH, and oxygen levels, and it has a complex filtration system that prevents the accumulation of metabolites, thereby maintaining physiological levels of these metabolites. TIM-2 is inoculated with a pooled microbiota to allow all experiments to start with the same starting microbiota [20]. After an adaptation period where the microbiota can get used to the new environment, an intervention can be performed, usually for a test period of 72 h. This allows for the study of the mycobiome and microbiome over a longer period of time than just a single time-point of a fecal sample, which could give more insights into the interactions between these kingdoms. The aim of this study therefore was to use TIM-2 to study fungal-bacterial interactions, and to investigate what happens if one or the other of these communities is disrupted.

## 2. Materials and Methods

### 2.1. TNO’s In Vitro Model of the Colon (TIM-2)

To mimic the realistic conditions of the colon, a sophisticated computer-controlled in vitro model (TIM-2) was used. The model is explained in detail in [17]. In short, in the model’s pH and temperature are controlled to resemble the circumstances in the colon of a healthy adult, at 5.8 and 37 °C, respectively. The pH is controlled by the addition of 2M NaOH. An anaerobic environment is created by a constant flush of N_2_ through the model and produced metabolites are removed with the use of a dialysis system. At the start of the experiment, the model is inoculated with a fecal pool mixed with a dialysis solution (explained below), which is the start of the adaptation period, to allow the microbiota to adapt to its new environment. The standard growth medium (described below) is added automatically at a constant rate. After the adaptation period, the test period is started where the different treatments are introduced. The model has been validated and extensively used for studying the bacterial component of the microbiota.

### 2.2. Fecal Pool

From healthy adult volunteers (*n* = 6, 50% female), fecal samples were collected and homogenized under anaerobic conditions. The samples were mixed, and after snap-freezing in liquid nitrogen, samples were stored at −80 °C until the start of the experiment. All experiments were performed with the same fecal pool. Preparation of the fecal pool is described in more detail in [20]. Before the start of the experiment, the tubes containing the fecal pool were thawed at a constant temperature (37 °C) in a water bath. Next, they were mixed 1:1 with dialysis solution that contained 2.5 g/L K_2_HPO_4_·3H_2_O, 4.5 g/L NaCl, 0.005 g/L FeSO_4_·7H_2_O, 0.5 g/L MgSO_4_·7H_2_O, 0.45 g/L CaCl_2_·2H_2_O, 0.05 g/L bile and 0.4 g/L cysteine-HCl, plus 1.5 mL vitamin mixture (containing 1 mg/L menadione, 2 mg/L D-biotin, 0.5 mg/L vitamin B12, 10 mg/L pantothenate, 5 mg/L nicotinamide, 5 mg/L p-aminobenzoic acid and 4 mg/L thiamine). This fecal-dialysate mixture was introduced anaerobically in the TIM-2.

### 2.3. Test Products

During the adaptation period, the standard growth medium was used as feeding. This is the simulated ileal efflux medium (SIEM), which was created to resemble the undigested dietary components that reach the colon [21], and contains 100 g CHO-medium (containing 12 g/L pectin, 12 g/L xylan, 12 g/L arabinogalactan, 12 g/L amylopectin, 100 g/L starch), 25 g TBCO 6.25× (containing 270 g/L Tween 80, 375 g/L bactopepton, 375 g/L casein, 6.25 g/L ox-bile), 2 g MgSO_4_ (50 g/L), 2 g cysteine (20 g/L), 0.2 mL vitamin mixture as described above, 4 mL salts solution (containing 4.7 g/L K_2_HPO_4_∙3H_2_O, 8.4 g/L NaCl, 0.8 CaCl_2_∙2H_2_O, 0.009 g/L FeSO_4_∙7H_2_O, 0.02 g/L haemin) and 1 mL antifoam emulsion. During the test period, SIEM was also used as growth medium, with the addition of fungicide or antibiotics. The fungicide used was cycloheximide, which was added as a shot (1.5 mg) at the start of the test period, and added to the SIEM (0.75 mg/day) and the dialysate (10 mg/L). As antibiotics, an equal mixture of ampicillin, oxytetracyclin and kanamycin was added at the same level as the fungicide, with in the shot a total of 1.5 mg antibiotics. The experiment with SIEM without any addition was run as a control.

### 2.4. Test Design

The experiment started with the inoculation of the fecal pool in the model, which marked the start of the adaptation period for 16 h. After the adaptation period, the test period was started, which lasted 72 h. Samples were taken from the lumen and the dialysate at the start of the experiment and every 24 h. The interventions of SIEM, SIEM+fungicide and SIEM+antibiotics were done in duplicate.

### 2.5. Gut Mycobiota and Microbiota Composition

For the determination of both the fungal and bacterial composition, DNA was isolated from TIM-2 samples. The DNA isolation was started with a bead-beating step as described before [22], in combination with the QIAamp Fast DNA stool mini kit from Qiagen (Venlo, The Netherlands). To measure the DNA concentration, the Qubit HS Assay and a Qubit 3.0 Fluorometer (Invitrogen, Waltham, MA, USA) were used.

For the mycobiota composition, the internal transcribed spacer unit 2 (ITS2) was sequenced with barcoding according to the Fungal Metagenomic Sequencing Demonstrated protocol of Illumina with some changes (Nextera XT DNA Library Preparation Kit, Nextera XT Index Kit v2 Set A, Illumina, Eindhoven, The Netherlands). Input DNA was 50 ng/µL and the PCRI program was set at 30 cycles. The primer set used was ITS F (5′-GCATCGATGAAGAACGCAGC-3′) and ITS R (5′-TCCTCCGCTTATTGATATGC-3′) [23]. Fragment sizes were analyzed using the Bioanalyzer with the DNA1000 kit (Agilent, CA, USA), and quantification, normalization, and equimolar pooling was done before loading the library on the Illumina Miseq system (Miseq reagent kit v3, Illumina). For the microbiota composition, the V3-V4 region of the 16S rRNA gene was sequenced according to the Illumina protocol with barcoding (Nextera XT DNA Library Preparation Kit, Nextera XT Index Kit v2 Set A, Illumina). The primer set used was 341F (5′-CCTACGGGNGGCWGCAG-3′) and 785R (5′-GACTACHVGGGTATCTAATCC-3′). The library was loaded on the Illumina Miseq system according to the manufacturer’s protocol (Miseq reagent kit v3, Illumina). Both libraries were sequenced on the Miseq system with the use of the Local Run Manager Generate FastQ Analysis Module v3 to generate fastq files. Further analysis of fastq files was performed with the Quantitative Insights Into Microbial Ecology 2 (QIIME2) software package (version 2019.7) [24,25]. For the ITS2 sequences, the QIIME2 plugin Q2-ITSxpress was used to trim ITS sequences [26]. Demultiplexing, quality filtering, and denoising was performed with the dada2 plugin [27]. The UNITE database (version 02-02-19) was used as reference database for the classification of ITS2 sequencing data. The SILVA database (version 132) was used as reference database for the classification of 16S rRNA sequencing data. QIIME2 was used to obtain alpha diversity indexes (observed features and effective Shannon diversity). Further analysis was done using Rstudio (R version 4.0.4) using the packages qiime2R, phyloseq, ggplot2, ggpubr, FSA and complexHeatmap. Groups were compared using the Kruskal–Wallis test, with Dunn’s test as post-hoc analysis. Beta diversity was visualized as Bray Curtis dissimilarity and Jaccard similarity and visualized as principal coordinate (PCoA) plots. Differences between groups were analyzed using PERMANOVA. GraphPad Prism version 9.3.0 was used for the visualization of SCFA data.

### 2.6. Short-Chain Fatty Acid (SCFA) Analysis

SCFAs (acetate, propionate, and butyrate) in the lumen and dialysate samples were analyzed with gas chromatography–mass spectrometry (GC-MS). Samples were prepared for GC-MS as described before [28]. In short, the samples were centrifuged and formic acid, 2-ethyl butyric acid (internal standard) and methanol were added to the supernatant. The analysis was carried out on a GC-MS (8890 GC System; Agilent Technologies, Amstelveen, The Netherlands) equipped with a PAL3 RSI 85 autosampler (Agilent) by injecting 1 μL sample on a DB-FATWAX Ultra Inert column (30 m, 0.25 mm, 0.25 μm, Agilent). The temperature settings of the injector port, oven, flame-ionization detector, and mass spectrometer detector were 250, 200, 275 and 225 °C, respectively. The flow rate over the column was 1.2 mL/min. With the use of calibration curves of known quantities of standards, quantities of SCFAs in the samples were determined.

## 3. Results and Discussion

**Figure 1:**
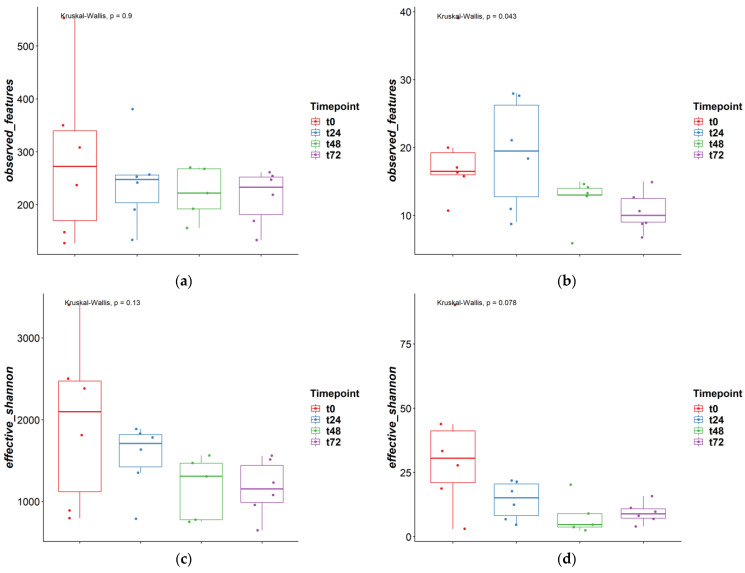
Alpha diversity measures different time points ‘observed features’ and ‘effective Shannon’; (**a**) and (**c**) = bacteria, (**b**) and (**d**) = fungi.

To investigate the fungal-bacterial interactions in TIM-2, the fungal and bacterial populations were analyzed on diversity, and perturbations were investigated when one of the populations was disrupted with the antimicrobials. In Figure 1, the alpha-diversity for the microbiota and mycobiota were compared for the different time points to see how time influences these diversity indexes and if this was different for bacteria and fungi. In Figure 1a,b, the observed features are shown at ASV level. As described before in other studies, the observed features for bacteria are much higher when compared to fungi, with a median of 242 and 14, respectively. In the fungal population, for observed features, a small decrease can be observed over time. Different time points were compared with the Kruskal-Wallis test and for fungi this change was significant (*p* < 0.05). Post-hoc Dunn’s test was performed and showed that t0 vs. t72 and t24 vs. t72 were significantly different. The effective Shannon index was also determined for both bacteria and fungi (Figure 1c,d), with a median of 1470 and 11, respectively, and this was also higher for bacteria compared to fungi. The effective Shannon did not change significantly between the different time points for bacteria and fungi.

**Figure 2:**
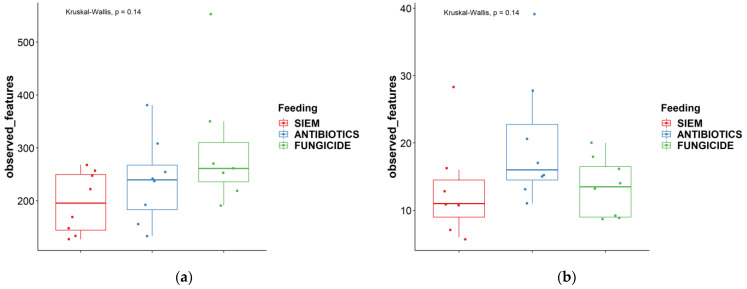

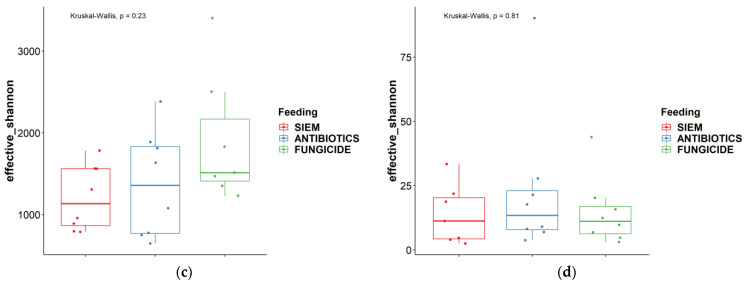
Alpha diversity measures different treatments ‘observed features’ and ‘effective Shannon’; (**a**) + (**c**) = bacteria, (**b**) + (**d**) = fungi.

Next to the comparison between time points, the alpha diversity was also compared between the different treatments. As can be seen in Figure 2, both the observed features and the effective Shannon did not change significantly between the different treatments.

**Figure 3:**
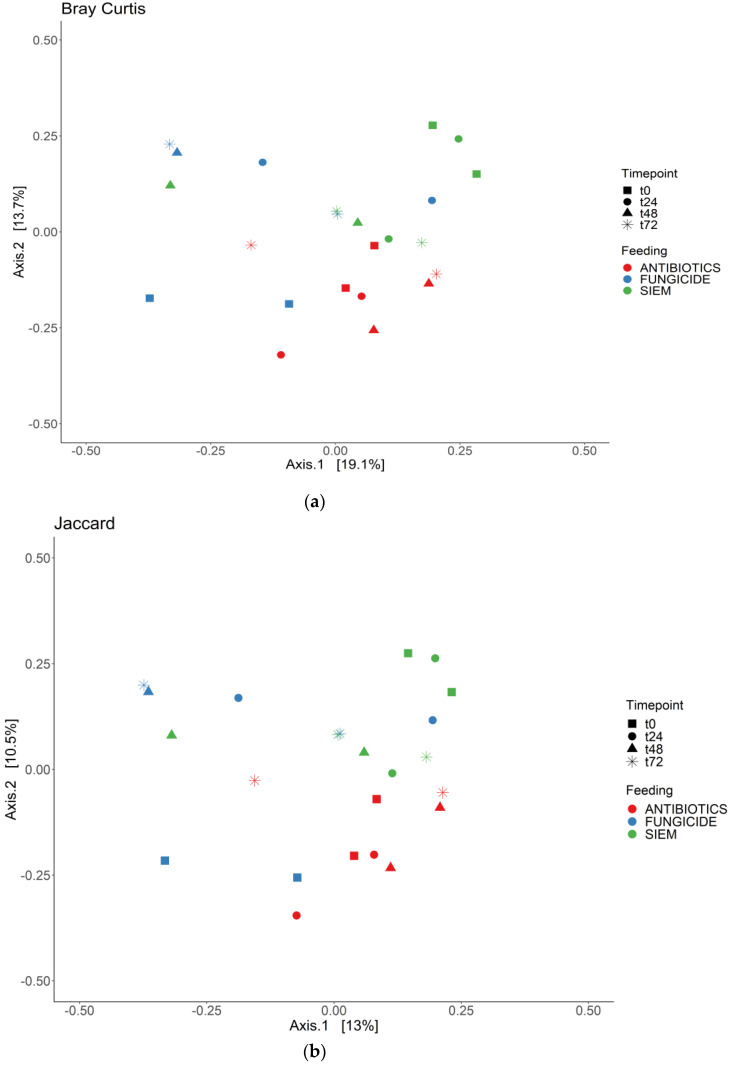
PCoA for bacteria; (**a**) Bray Curtis dissimilarity; and (**b**) Jaccard.

**Figure 4:**
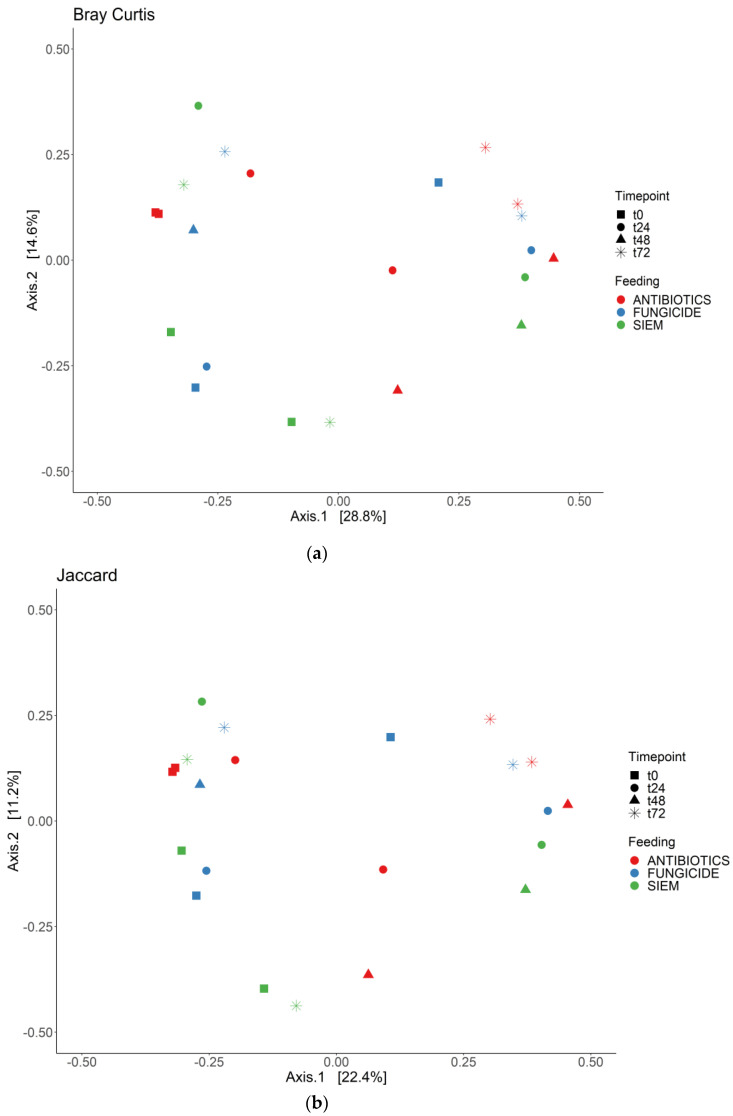
PCoA for fungi; (**a**) Bray Curtis dissimilarity; and (**b**) Jaccard.

To see if the samples would cluster together for the different time points or the treatments, the beta diversity was analyzed for both bacterial and fungal communities. The Bray Curtis dissimilarity and Jaccard similarity scores for bacteria are shown as a PcoA plot in Figure 3a,b. It can be observed that the samples that were treated with antibiotics cluster together, whereas the samples with SIEM and fungicide as intervention were more spread out. The Bray Curtis dissimilarity scores for the different inventions were compared using PERMANOVA, and samples treated with antibiotics were significantly different compared to both other inventions (*p* < 0.05). For the Jaccard index, all pairwise comparisons were significant (*p* < 0.05). The t0 samples treated with antibiotics and fungicide are more close together, after which they move away from each other when the experiment continues. From the Bray Curtis and Jaccard indexes for fungi (Figure 4a,b), clustering for the different treatments was less clear. The Bray Curtis dissimilarity scores were not significantly different when compared (PERMANOVA), but the pairwise comparison of the Jaccard index between the fungicide and the antibiotic treatment was significant (*p* < 0.05). For the different time points, there is variation observed. This greater variability in the mycobiome is similar to what was found before.

Antibiotic treatment has been linked to the disruption of the commensal bacterial community found in the gut, which could lead to a reduction in beneficial species and an increase in antibiotic-resistant and/or pathogenic bacteria [29]. This is especially the case when broad-spectrum antibiotics are used that can affect a wide range of Gram-negative and Gram-positive bacteria. Antibiotic treatment has also been shown to affect the fungal community found in the gut. Treatment with antibiotics can be followed by an increase in fungal species, which can lead to fungal infections [30,31]. These data suggest that bacteria can have an inhibitory effect on the growth of fungi. This could be because they compete for nutrients, but also metabolites produced by bacteria could have an effect on the mycobiota [32,33,34]. These findings are confirmed in a mouse model, where normal mice were more resistant to pathogenic fungi than mice treated with antibiotics [35]. It was also shown that mice treated with antibiotics showed significant changes in their gut microbiota, and it takes a considerable amount of time for the microbiota to return to the state before the treatment [36,37].

The effect of anti-fungal treatment on gut bacterial and fungal species is less investigated. Human data is scarce, but several studies on mice show that anti-fungal treatment could have an effect on the bacterial component of the gut microbiota. In a mouse model where mice were treated with anti-fungal treatment, the fungal diversity was decreased, whereas bacterial diversity was increased compared to controls [14]. Another study in mice showed that mice treated with anti-fungal drugs showed changes in the bacterial composition [38]. The introduction of five species of fungi in gnotobiotic mice induced alterations in the gut bacteria [39].

**Figure 5:**
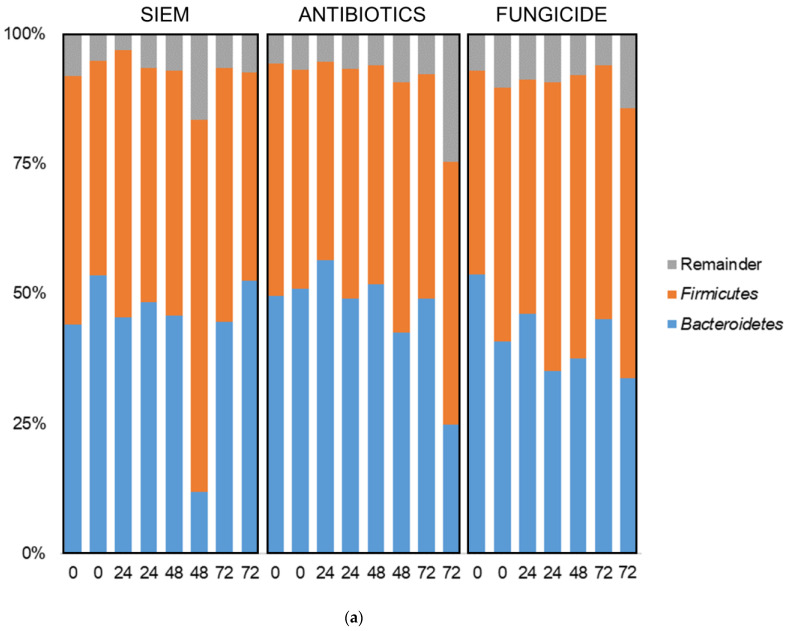

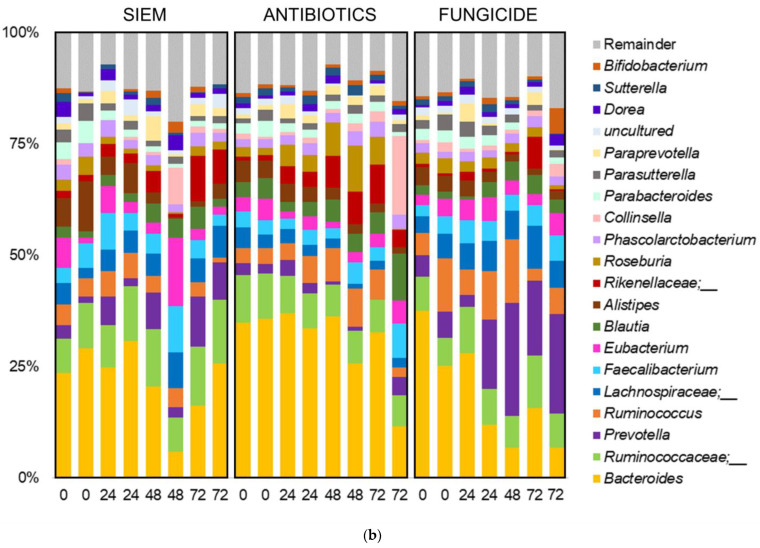
Taxonomic classification of bacteria at (**a**) phylum and (**b**) genus level.

In addition to the diversity analyses, taxonomic classification of samples was also done. In Figure 5, the bacterial relative abundance for the different samples is shown at the phylum and genus level. The most abundant phyla found in all samples were *Bacteroidetes* and *Firmicutes* (Figure 5a). No big shifts could be seen for the different treatments. The 20 most abundant genera are also shown; in these genera also, no significant differences between the treatments were observed (Figure 5b). Other studies on the effect of fungicides on the microbiota do describe an effect. In a study on the effect of oral exposure to the fungicide carbendazim, disturbances were observed in mice treated with this fungicide. A reduction of the relative abundance of *Bacteroidetes* and an increase of *Firmicutes*, *Actinobacteria*, and *Proteobacteria* was found [40]. In another study on mice, on the effect of exposure to propamocarb on gut microbiota, changes were observed. The relative abundances of the genera *Oscillospira*, *Parabacteroides*, *Desulfovibrio*, *Ruminococcus*, *Bacteroides*, *Dehalobacterium*, *Butyricimonas*, *Prevotella*, and *Dorea* were different after exposure to the fungicide [41]. Here, cycloheximide was used, which may have led to different results.

**Figure 6:**
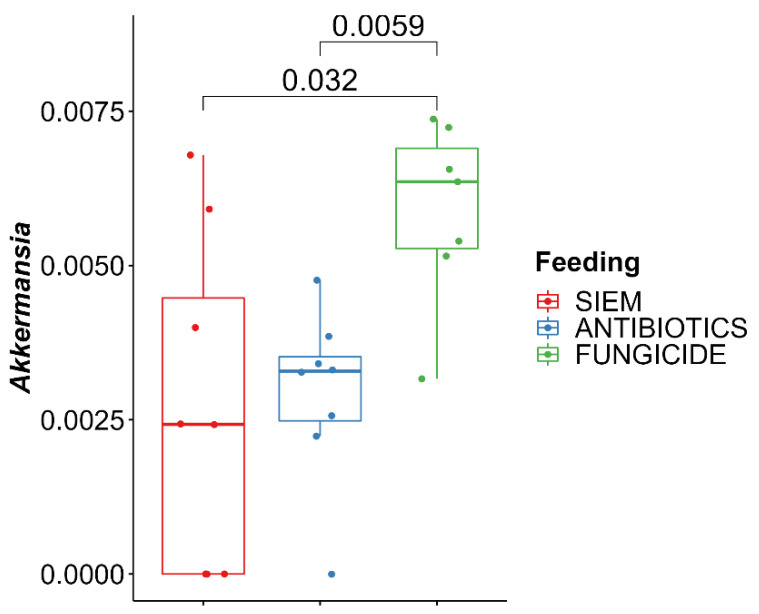
Kruskal–Wallis with Dunn’s post-hoc pairwise comparison for *Akkermansia*.

With the use of a Kruskall–Wallis test, significant differences for the genera were checked between the different treatments. From this, it was shown that *Akkermansia* was higher in the samples treated with fungicide (Figure 6). The increase of *Akkermansia* after fungicide treatment was also seen in mice [42]. Here, mice were treated with tebuconazole, after which significant changes in the gut microbiota were observed, with in particular an increase in *Akkermansia*. *Akkermansia* is found in the outer mucus layer of the gut and plays an important role in maintaining the mucus layer [43]. In addition, it regulates tight junction proteins, thereby regulating the intestinal barrier function [44]. A reduction in *Akkermansia* levels could have an effect on the barrier of the intestine and could thereby induce colonic inflammation.

**Figure 7:**
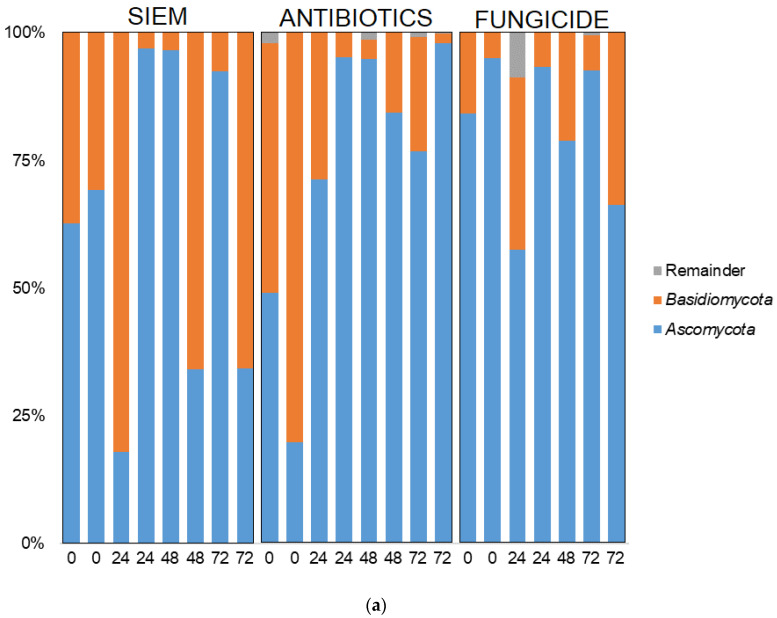

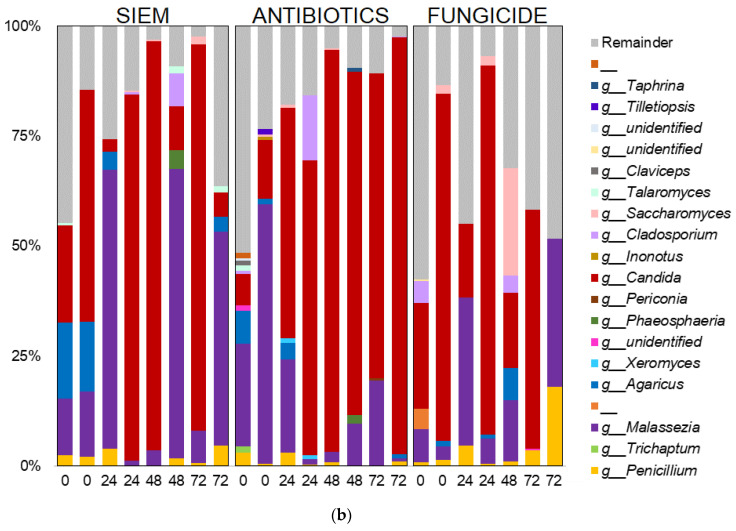
Taxonomic classification of fungi at (**a**) phylum and (**b**) genus level.

Taxonomic classification was also done for the mycobiota (Figure 7). Here, the phyla *Ascomycota* and *Basidiomycota* were most abundant, but some variation in the ratio between the two phyla can be seen (Figure 7a). The 20 most abundant genera are shown in Figure 7b. The dominance of *Candida* can be observed in several samples, but especially in the treatment with antibiotics, this genus becomes dominant over the course of the experiment. The outgrowth of *Candida* after antibiotic treatment is also described in literature. E.g., Gutierrez et al. describe the colonization of *C. albicans* after broad-spectrum use of antibiotics in mice [45]. Gut bacteria can have an influence on fungal proliferation of *Candida* or other fungi by the production of metabolites, e.g., cell wall components, thereby directly influencing the fungi. The effect could also be indirect, where bacteria influence host responses, which in turn affect fungal growth [46]. An example of how bacterial metabolites can influence fungi is the promotion of *C. albicans* hyphal growth after antibiotic treatment in mice, caused by the release of bacteria peptidoglycan in the gut lumen [47]. In another study, the proliferation of *C. albicans* was inhibited in a continuous-culture bioreactor system, when bacterial metabolites derived from 50 strains from human fecal samples were added [33].

**Figure 8:**
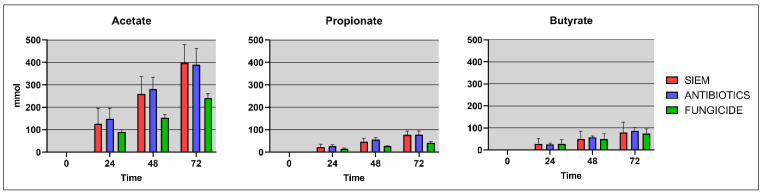
Cumulative SCFA levels (mmol/L) for the different treatment groups.

Moreover, short-chain fatty acids (SCFAs) have shown to have an effect on fungal growth, therefore next to the gut microbiota, the SCFAs (acetate, propionate and butyrate) were also determined in the lumen and dial samples of the TIM-2 experiments. These SCFA levels were compared for the different treatments (Figure 8).

As has been described in the literature, mainly acetate was found in the samples, along with smaller amounts of propionate and butyrate. The disruption of the bacterial community after fungicide treatment can lead to altered SCFA levels in the gut. In line with this, levels found in the treatments SIEM and antibiotics were slightly higher when compared with fungicide treatment. Wu et al. found that after exposure with the fungicide propamocarb, the SCFA propionate and the BCFA isobutyrate were significantly increased in fecal samples of mice [41]. Research on the effect of fungicides on SCFAs is scarce. The effect of antibiotic use on SCFA production was previously studied using TIM-2, where the SCFA levels were also not effected, similar to what was observed in the current study [48]. The interplay between bacteria, fungi, and SCFAs should be further investigated to get more insights into these processes. There are some examples where SCFAs were shown to have an inhibitory effect on fungal growth. For instance, SCFAs have been shown to have an inhibitory effect on *C. albicans* growth in vitro by influencing germ tube formation and a reduction of fungal metabolic activity in biofilms [49].

**Figure 9:**
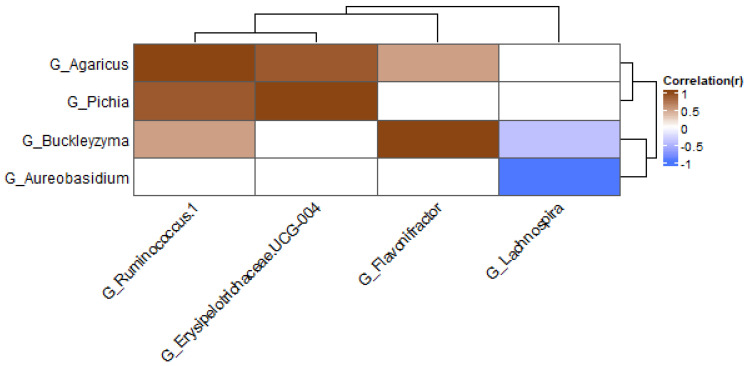
Heatmap at genus level for antibiotics samples; only genera present in at least 20% of samples were included.

**Figure 10:**
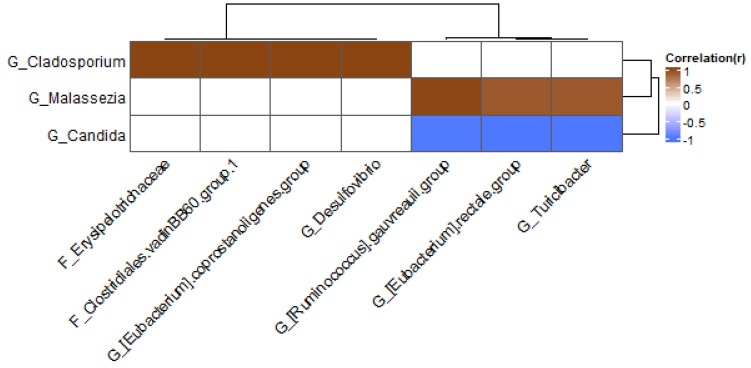
Heatmap at genus level for fungicide samples; only genera present in at least 20% of samples were included.

The results above suggest that interactions between bacteria and fungi are present in the gut. Correlations were studied between bacteria and fungi to see if interactions between specific species were present. The correlations were performed in the groups with the different treatments. The correlations between fungal and bacterial genera are shown in Figure 9 and Figure 10. In the samples treated with antibiotics, several correlations between genera were found (when these were present in at least 20% of samples; Figure 9). The fungal genera *Agaricus* and *Pichia* were positively correlated with *Ruminococcus* and *Erysipelotrichaceae*. One of the members of the genus *Agaricus* is *A. bisporus*, also known as the white mushroom. Members of this genus possibly have prebiotic properties [50]. In pigs fed with *Agaricus*, levels of *Ruminococcaceae* were increased, suggesting that *Agaricus* can have a positive effect on the growth of these bacteria [51]. The family *Erysipelotrichaceae* is commonly described as an inhabitant of the human gut, and in this study, it is positively correlated with the fungal genus *Pichia*. Previous research shows that this bacteria can potentially grow out after treatment with broad-spectrum antibiotics [52]. The fungal genus *Aureobasidium* was shown to be negatively correlated with *Lachnospira* in the group treated with antibiotics. *Aureobasidium* is a yeast that can be found in diverse habitats. They show antimicrobial activity against bacteria and fungi and are therefore used in agriculture and industry [53]. The abundance of *Lachnospira* is positively correlated with the consumption of fruits and fiber [54]. They are known fiber and pectin degraders, and with the products released with this degradation, *Lachnospira* can influence the growth of other bacteria via cross-feeding [55,56].

In the group treated with fungicide, the fungal genus *Cladosporium* was positively correlated with several bacteria (Figure 10): *Erysipelothrichaceae*, *Clostridiales* vadin BB60 group, *Eubacterium coprostanoligenes* group, and *Desulfovibrio*. The fungus *Malassezia* was positively correlated with the *Ruminococcus gauvreauii* group, *Eubacterium rectale* group, and *Turicibacter*, whereas *Candida* was negatively correlated with these bacteria.

These correlations were found after treatment with antibiotics or fungicide and not in the samples without treatment. This suggests that these treatments can cause a shift in the microbiota. The correlations that were found in this study should be explored further to see if they have biological relevance and if they can be replicated in vivo. More in vitro experiments could help in unraveling mechanisms.

## 4. Conclusions

In conclusion, in the experiment with human fecal samples performed in TIM-2 where the microbiota was treated with antibiotics or fungicide, the bacterial communities were more diverse compared to fungi. No significant differences in alpha diversity were observed between the different treatment groups. From the beta diversity analyses, it could be observed that samples treated with antibiotics clustered together, whereas SIEM and fungicide samples were more spread. Taxonomic classification of both bacteria and fungi was performed, but no big shifts were seen after treatment. The bacteria Akkermansia was increased after fungicide treatment. SCFAs levels were determined, and these were slightly lower in samples treated with fungicide. Correlations between fungi and bacteria were made. These correlations suggest that cross-kingdom relations are present and that they can influence each other. Further research is needed to gain more insight into these relationships. For instance, absolute numbers of taxa should be evaluated rather than just relative abundance to see if the total (viable) count of bacteria and/or fungi changed upon antimicrobial treatment.

## Data Availability

The raw sequences and corresponding metadata will be archived in the Sequence Read Archive (SRA) repository at the NCBI upon acceptance of the manuscript: http://www.ncbi.nlm.nih.gov/bioproject/902713 and http://www.ncbi.nlm.nih.gov/bioproject/907310.
